# Using microarray‐based subtyping methods for breast cancer in the era of high‐throughput RNA sequencing

**DOI:** 10.1002/1878-0261.12389

**Published:** 2018-10-29

**Authors:** Christina Bligaard Pedersen, Finn Cilius Nielsen, Maria Rossing, Lars Rønn Olsen

**Affiliations:** ^1^ Department of Bio and Health Informatics Technical University of Denmark Kemitorvet, Kongens Lyngby Denmark; ^2^ Center for Genomic Medicine Rigshospitalet ‐ Copenhagen University Hospital Denmark

**Keywords:** breast cancer, gene expression, molecular subtyping, RNA sequencing

## Abstract

Breast cancer is a highly heterogeneous disease that can be classified into multiple subtypes based on the tumor transcriptome. Most of the subtyping schemes used in clinics today are derived from analyses of microarray data from thousands of different tumors together with clinical data for the patients from which the tumors were isolated. However, RNA sequencing (RNA‐Seq) is gradually replacing microarrays as the preferred transcriptomics platform, and although transcript abundances measured by the two different technologies are largely compatible, subtyping methods developed for probe‐based microarray data are incompatible with RNA‐Seq as input data. Here, we present an RNA‐Seq data processing pipeline, which relies on the mapping of sequencing reads to the probe set target sequences instead of the human reference genome, thereby enabling probe‐based subtyping of breast cancer tumor tissue using sequencing‐based transcriptomics. By analyzing 66 breast cancer tumors for which gene expression was measured using both microarrays and RNA‐Seq, we show that RNA‐Seq data can be directly compared to microarray data using our pipeline. Additionally, we demonstrate that the established subtyping method CITBCMST (Guedj *et al*., 2012), which relies on a 375 probe set‐signature to classify samples into the six subtypes basL, lumA, lumB, lumC, mApo, and normL, can be applied without further modifications. This pipeline enables a seamless transition to sequencing‐based transcriptomics for future clinical purposes.

**Abbreviations**
CITBCMST: CIT breast cancer molecular subtypes
FPKM: fragments per kilobase per million
MAD: median absolute deviation
PCA: principal component analysis
PREBS: probe region expression estimation based on sequencing
QC: quality control
RMA: robust multi‐array average
RNABC: RNA breast cancer
RNA‐Seq: RNA sequencing
SCC: Spearman's rank correlation coefficient
TDM: training distribution matching
TPM: transcripts per million

## 1 Introduction

Breast cancer is a highly heterogeneous disease with several clinical subtypes defined by transcriptomic expression profiles that correlate with pathogenesis, clinical features, and prognosis (Goldhirsch *et al*., 2013; Parker *et al*., 2009; Stratton *et al*., 2009). Multiple studies have defined molecular classification schemes for breast cancer (Cronin *et al*., 2007; Guedj *et al*., 2012; Sorlie *et al*., 2003; van de Vijver *et al*., 2002), most of which are based on transcript quantification measured by bead‐ or probe‐based microarrays. The use of DNA microarrays has been pivotal to cancer research for the past decades, but transcriptomics is moving toward RNA sequencing (RNA‐Seq) as this technique allows for quantification of previously uncharacterized transcripts, as well as novel genetic aberrations such as fusion genes and alternative splicing (Soneson and Delorenzi, 2013; Vitting‐Seerup and Sandelin, 2017; Zwiener *et al*., 2014). However, due to the abundance of useful and widely employed probe‐based subtyping tools, the implementation of RNA‐Seq in clinical settings is falling behind the implementation in the general research community (Thompson *et al*., 2016).

Comparisons of microarray and RNA‐Seq transcriptomics have shown that the results of the two techniques are comparable in a general sense and yield similar results in nonparametric analyses (Chen *et al*., 2017; Fumagalli *et al*., 2014; Marioni *et al*., 2008; Uziela and Honkela, 2015), indicating potential for RNA‐Seq to eventually replace microarrays for molecular subtyping. The primary obstacle for the transition to RNA‐Seq transcript quantification in microarray‐based subtyping schemes is the fact that the nature of the data from these two methods differs vastly. Because most classification algorithms assume that training and test data are drawn from the same distribution, a so‐called dataset shift occurs when the RNA‐Seq data are submitted directly to algorithms trained on probe‐based expression data. For RNA‐Seq data to be useful for subtyping using algorithms trained on microarray data, it is therefore necessary that the data are made comparable in the strictest sense. In order to facilitate a shift toward sequencing‐based transcriptomics in our clinic, we developed an RNA‐Seq data processing pipeline that makes these data compatible with existing probe‐based subtyping methods. We compared our method to three previously reported methods for comparing RNA‐Seq‐based transcriptomics data to microarray‐based transcriptomics data: (a) direct comparison of fragments per kilobase per million (FPKM) with probe intensities summarized to transcript level (Chen *et al*., 2017; Fumagalli *et al*., 2014), (b) Training Distribution Matching (TDM) (Thompson *et al*., 2016), and (c) Probe Region Expression estimation Based on Sequencing (PREBS) (Uziela and Honkela, 2015). We show that our method outperforms existing methods and enables direct application of RNA‐Seq data for molecular subtyping of breast cancer. The method is, in principle, compatible with all microarray‐based cancer subtyping methods.

## 2 Materials and methods

### 2.1 Assigning subtypes to samples using CITBCMST

The CIT Breast Cancer Molecular SubTypes (CITBCMST) subtyping method employed at our clinic is a machine learning‐based model constructed on 355 selected samples from primary breast carcinomas, which were collected in France in the Cartes d'Identité des Tumeurs (CIT) program and analyzed on Affymetrix HG‐U133 Plus 2.0 arrays (Guedj *et al*., 2012). Using these data, the authors defined a 375 probe set‐signature and six distinct molecular subtypes, basL, lumA, lumB, lumC, mApo, and normL, and provided a script to assign one of the six subtypes to new samples profiled using a microarray (Affymetrix HG‐U133 Plus 2.0 or similar). The CITBCMST method classifies samples based on the intensity of the 375 probe sets using a distance‐to‐centroid approach, where each sample is assigned to the subtype with the closest centroid (per default based on diagonal linear discriminant analysis). Alternatively, CITBCMST can classify samples using a 256 HUGO gene symbol signature summarized from the 375 probes, in which case the distance to each centroid is calculated as (1—Pearson correlation coefficient). No co‐normalization or batch correction is implemented in the CITBCMST algorithm, but we routinely include these transformations before classification in clinical practice (Rossing *et al*., 2018).

In several parts of the data processing in this study, we applied the CITBCMST training dataset; the full dataset (537 CEL files) was downloaded from ArrayExpress (accession: E‐MTAB‐365), preprocessed as described by the authors, and reduced to the 355 core samples used to train the model. We also removed the AFFX control probe sets before any further application.

In order to visualize the subtype calls, CITBCMST produces two principal component analysis (PCA) plots of PCs 1 and 2: one for the training data and one for the test samples. In this study, PCA plots have been generated using a slightly modified version of the CITBCMST source code, producing a single plot containing both the training data and the classified samples. Furthermore, we modified the CITBCMST script to perform the PCA on the training data and then subsequently projecting new data into this PC space by scaling and multiplying the vectors with the rotation, in order to produce comparable PCA plots for each run of the script.

### 2.2 Patients, tumor samples and RNA isolation

The test data for this study are comprised of two datasets: one generated at the Breast Cancer Translational Research Laboratory, Institut Jules Bordet, in Brussels, Belgium, published in Fumagalli *et al*. (2014) consisting of paired microarray and RNA‐Seq data measured in tumors from 57 breast cancer patients, and one set consisting of nine tumors for which paired microarray and RNA‐Seq data were generated at Rigshospitalet (RH), Copenhagen University Hospital, Denmark.

In the former dataset (henceforth referred to as the ‘Bordet’ dataset), the samples comprise a balanced mix of the four IHC breast cancer subtypes: 17 triple negative, 14 HER2 positive, 16 luminal A, and 10 luminal B patients. In this work, we reclassified the Bordet samples using the CITBCMST six‐class subtyping scheme (Guedj *et al*., 2012).

In the latter dataset (henceforth referred to as the ‘RH’ dataset), tumor specimens originated from nine women diagnosed with breast cancer undergoing primary surgical procedures (during 2015 and 2016) at RH. The study was approved by The Danish Data Protection Agency (jr. no.: 2012‐58‐0004) and Danish Breast Cancer Group (jr. no.: DBCG‐2015‐14), meaning that tumor material was obtained with the informed consent of the patients and the study conforms to the standards established by the Declaration of Helsinki. Following surgical resection, fresh tumor specimens were evaluated by designated pathologists and tumor biopsies (~ 100 mg) were stored in RNAlater (Thermo Fisher Scientific, Waltham, MA, USA). RNA was isolated using the AllPrep DNA/RNA purification kit (Qiagen, Hilden, Germany) and the QIACube workstation according to the manufacturer's instructions. The integrity of the RNA was measured using the Agilent RNA 6000 Nano Kit on an Agilent 2100 Bioanalyzer (Agilent Technologies, Inc., Santa Clara, CA, USA).

### 2.3 Microarray analysis

For the Bordet dataset, the transcriptomic profiles were obtained using the Affymetrix HG‐U133 Plus 2.0 microarray. The raw Affymetrix cell intensity files (.CEL files) files are available on the NCBI Gene Expression Omnibus under accession number GSE43358.

To generate the RH dataset, RNA was reverse transcribed and used for cRNA synthesis, and labeling and hybridization with the Affymetrix HG U133 Plus 2.0 microarray were carried out according to the manufacturer's protocol. The arrays were washed and stained with phycoerythrin‐conjugated streptavidin using the Affymetrix Fluidics Station 450, and arrays were scanned in the Affymetrix GeneArray 3000 7G scanner. CEL files were generated in the GeneChip Command Console Software (agcc; Affymetrix, Thermo Fisher Scientific). For both datasets, quality control (QC) was performed using the R/Bioconductor package affyQCReport. After passing QC, the raw. CEL‐files were processed using robust multi‐array average (RMA) from the R/Bioconductor package affy (Gautier *et al*., 2004) unless otherwise indicated. Finally, the data were reduced to contain only the 54 613 noncontrol probe sets.

### 2.4 RNA sequencing

The Bordet dataset was sequenced on the Illumina HiSeq 2000 (Illumina, Inc., San Diego, CA, USA) as described in (Fumagalli *et al*., 2014) and was archived at the European Genome‐phenome Archive (EGA) under accession number EGAD00001000627.

RNA sequencing for the RH dataset was done using TruSeq Stranded Total RNA Library Prep Kit, and RNA was paired‐end sequenced on a NextSeq500 (Illumina, Inc.) to gain an average output of 50–100 m reads. Raw sequencing data from the Illumina sequencing platforms were processed with CASAVA‐1.8.2. FastQC (Andrews, 2010) was run on all samples to ensure a proper quality before further processing (Conesa *et al*., 2016).

### 2.5 Estimating target sequence abundance from RNA sequencing reads

#### 2.5.1 Fragments per kilobase per million

Raw reads were trimmed using Trimmomatic (Bolger *et al*., 2014) with settings LEADING:3 TRAILING:3 SLIDINGWINDOW:4:15 MINLEN:36. The trimmed reads were mapped to the human reference genome hg19 using TopHat2 (v. 2.1.1) (Kim *et al*., 2013), and Cufflinks (v. 2.2.1) (Trapnell *et al*., 2010) was used for gene expression quantification. Both processes used the annotation file (.GTF) from Ensembl (Zerbino *et al*., 2018). FPKM values for all genes were extracted from the genes.fpkm_tracking files and merged to a table for all samples, and FPKMs for duplicate genes were summed using ddply from the r package plyr (Wickham, 2011). This resulted in a total of 63 657 transcripts, for which the FPKM values were log‐transformed using the formula FPKM^′^ = log_2_(FPKM + 1).

#### 2.5.2 Transcripts per million

Reads were mapped using kallisto (Bray *et al*., 2016), which produces transcripts per million (TPM) as units of abundance, by estimating the proportion of reads mapping to the target sequence. Briefly, kallisto utilizes so‐called pseudoalignment, which is based on exact matching of k‐mers derived from reads, rather than traditional sequence alignment. This speeds up read mapping significantly and in some cases provides more accurate mapping than traditional approaches. The applied reference was the HG‐U133 Plus 2.0 Target Sequences, originally retrieved from http://www.affymetrix.com (a copy of this file as well as the mapping index file is available on https://bitbucket.org/cbligaard/rnabc/downloads). Each target sequence represents a probe set on the microarray, and individual probes in a given probe set are selected as subsequences of the target sequence.

### 2.6 Comparing microarray and RNA sequencing data

According to the CITBCMST classification protocol, the optimal data input is RMA normalized microarray data from an Affymetrix platform. However, the consensus on best practices handling of microarray data suggests normalization of intensity distributions and correction of batch effects between runs from different instruments, times, or operators (Johnson *et al*., 2007). In clinical practice, we routinely include these transformations before classification (Rossing *et al*., 2018). In this study, we compared four different processing pipelines for making subtyping results from the microarray and RNA‐Seq platforms comparable—three of these were previously published, and in those cases, the processing described by the original authors was followed as closely as possible, despite this meaning that microarray data were not always processed according to the described best practices.

#### 2.6.1 Direct comparison of transcript‐summarized probe intensities with transcript FPKM (Fumagalli)

Both data types were processed as described by Fumagalli *et al*. (2014). For microarray data, frozen RMA was applied using the R/Bioconductor package frma (McCall *et al*., 2010). To allow for direct comparison to RNA‐Seq expression levels, jetset (Li *et al*., 2011) was used to map between HUGO symbols and probe set IDs. If multiple probe sets mapped to the same gene, the one with the highest jetset score was chosen. For RNA‐Seq, starting with log‐transformed FPKM values, a translation of Ensembl gene IDs to HUGO symbols was performed using BioMart (Durinck *et al*., 2005). If a single HUGO symbol matched more than one Ensembl gene ID, the sum of the values for the corresponding gene was used. The CITBCMST classifier was then applied on the HUGO symbols (238/256 HUGO symbols were accepted by CITBCMST).

#### 2.6.2 Training Distribution Matching (TDM)

The TDM algorithm (Thompson *et al*., 2016) was developed to enable comparison of sequencing‐based and probe‐based transcriptomics data. Briefly, TDM leaves between‐sample relationships intact, but transforms the distribution to be similar to that of the training data. The rank order of most genes remains the same in order to not affect any biological significance found herein. The distribution is adjusted for an entire test dataset, in order to avoid overnormalization from adjusting per sample, and it includes a log_2_ transformation as microarray data are typically transformed as such.

The RNA‐Seq data were processed as described above, except that we utilized untransformed FPKM values as described by Thompson *et al*. (2016). We used the same HUGO translation from the Ensembl IDs and then used jetset on the full CITBCMST training dataset and the Bordet arrays to get corresponding genes before running TDM against the translated ‘full’ CITBCMST training dataset (18 734 genes included). We then performed CITBCMST classification on the RNA‐Seq data using 234 HUGO symbols and directly on RMA normalized microarray data. The correlation of transcript abundance was calculated using the full set of matching genes with the Bordet data.

#### 2.6.3 Probe Region E0xpression estimation Based on Sequencing (PREBS)

Briefly, the PREBS pipeline utilizes the mapped reads from each file stored in BAM format generated by standard alignment algorithms such as TopHat. PREBS then counts the number of reads overlapping transcript regions corresponding to probe sequences in order to estimate probe region expression (Uziela and Honkela, 2015). Microarray data were simply RMA normalized before subtyping.

#### 2.6.4 Mapping RNA‐Seq reads directly to microarray probe sequences (RNABC)

Reference transcripts are commonly extracted from a reference genome such as hg19, but instead, we used the target sequences of the Affymetrix HG‐U133 Plus 2.0 array as reference, such that the abundance estimates from RNA‐Seq data can be compared directly to the intensities from specific probes on the microarray platform. After removing control probe sets, ‘normalize.quantiles.target’ from the R/Bioconductor package ‘preprocessCore’ was used to quantile normalize the probe set sequence‐aligned TPM units using the distribution of the mean intensity of all probes in the full CITBCMST training dataset as the target distribution. After quantile normalization, ‘ComBat’ (Johnson *et al*., 2007) (from the R/Bioconductor package ‘sva’) was applied to remove batch effects between the CITBCMST training dataset and the test data. We named our pipeline RNA Breast Cancer (‘RNABC’). This pipeline was followed by CITBCMST subtyping on the probe set‐level. For consistency, we treated the microarray data in the exact same manner.

### 2.7 Performance metrics

The CITBCMST predictor produces a confidence score for each sample assignment: If a sample is close to several centroids, that is, the difference in distance from a sample to two or more of the centroids is smaller than the tenth percentile of the distances between the centroids in the training dataset, the score is set to ‘mixed’. If this is not the case, the score is set to either ‘core’ or ‘outlier’ depending on the distance to the closest centroid. If the distance is *n* times larger than the median absolute deviation (MAD) between the training data and the centroid for the given subtype, the sample is classified as an ‘outlier’. *n* is calculated as the maximum between the six subtypes of the value (max_distances to centroid_ ‐ med_distances to centroid_)/mad_distances to centroid_ (Guedj *et al*., 2012).

Performance was assessed by the number of perfect matches between the microarray predicted subtypes and the RNA‐Seq predicted subtypes, and the number of mismatches. We consider two types of mismatches: confidence mismatch (between ‘core’, ‘outlier’, or ‘mixed’ confidence label) and class mismatch (between either one of the subtypes)—the latter of course being the more severe error.

Additionally, we calculated the *R* ^2^ value for the least squares regression for microarray probe set intensities vs. RNA‐seq FPKM/TPM and the Spearman's Rho for a rank‐based measure of correlation, as we cannot expect probe set intensities and FPKM/TPM to correlate linearly for all preprocessing methods.

## 3 Results

### 3.1 Transformation of RNA sequencing data for use with the CITBCMST classifier

**Figure 1:**
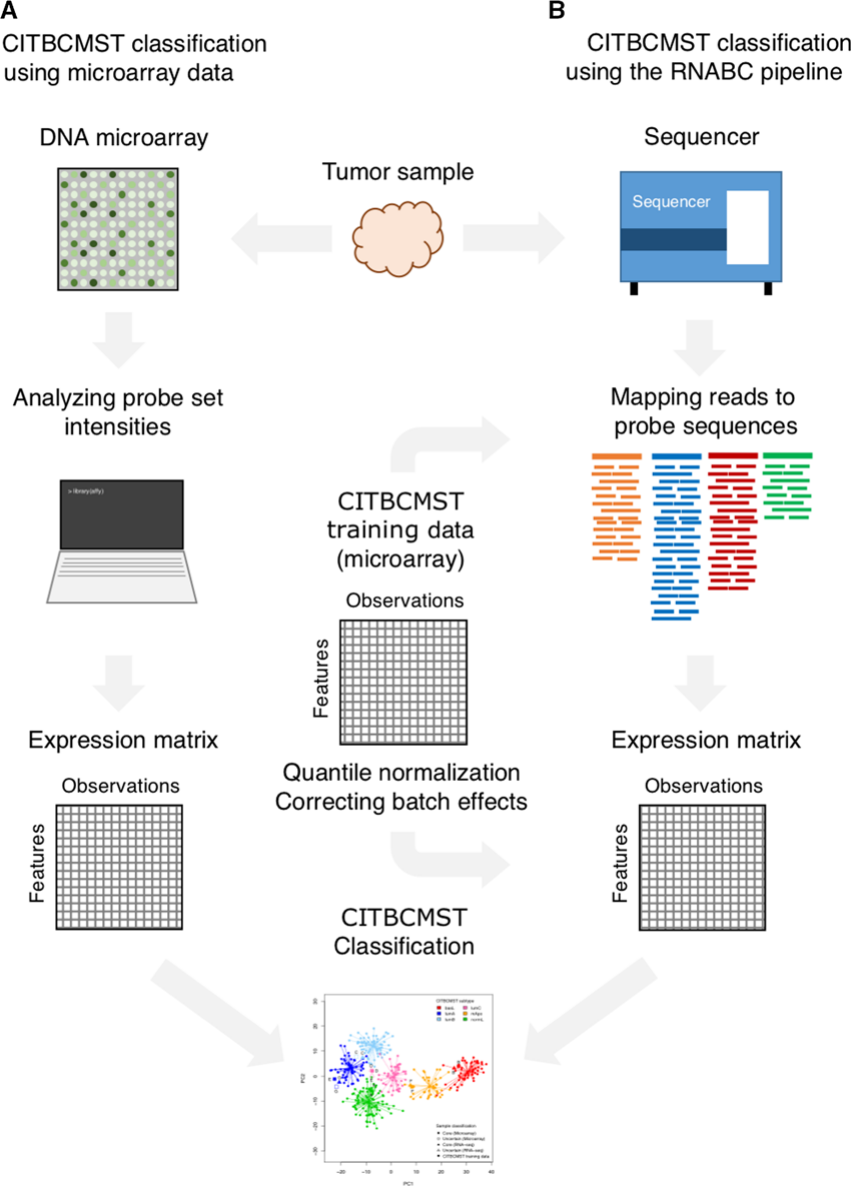
Pipelines for using CITBCMST classifier using (A) microarray data and (B) RNA‐Seq data with the RNABC pipeline. For microarray data, processed samples are submitted to the CITBCMST R package and the resulting subtype predictions are returned. In the RNABC pipeline, raw RNA‐Seq data are submitted, reads are mapped to probe target sequences using kallisto, read counts are quantile normalized and batch corrected using the CITBCMST training data (*n* = 355) as comparison, and classification is done on transformed counts.

In order to define the most accurate processing procedure, we tested multiple combinations of RNA‐Seq metrics, normalization, and batch correction—including existing methods for RNA‐Seq and microarray data comparison. We found that mapping RNA‐Seq reads to the array probe set sequences (rather than a reference transcriptome), followed by quantile normalization to a target distribution consisting of the mean probe intensities in the microarray training data, followed by batch correction with the microarray training data (Fig. 1) resulted in a very high correlation between microarray‐ and RNA‐Seq‐based abundances (*R* ^2^ = 0.9445 and Spearman's ρ = 0.9638) and highly similar subtyping (identical for 51 of the 57 samples) compared with the subtyping on the corresponding microarray data. The RNABC pipeline is implemented in the r programing language (R Core Team, 2018) and available at https://bitbucket.org/cbligaard/rnabc/.

### 3.2 Validation of the RNABC pipeline on the Bordet dataset

**Figure 2:**
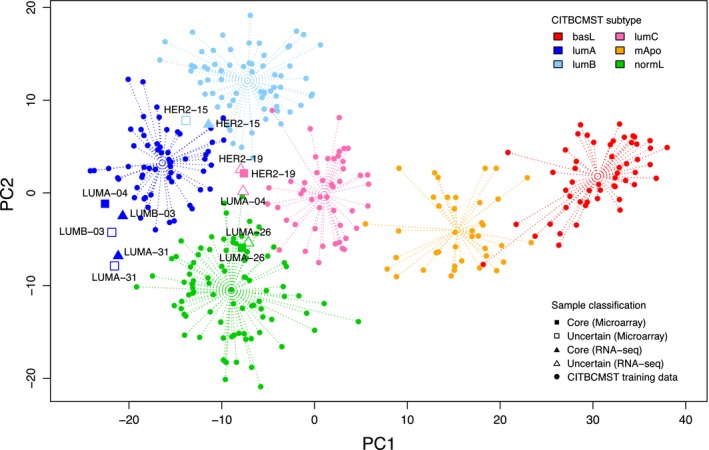
PCA plot of the CITBCMST training data (circles) and the six discordantly classified samples of the Bordet dataset from both microarray (squares) and RNA‐Seq (triangles) data. Each sample point is labeled by the original sample name from Fumagalli *et al*. (2014) to allow for direct comparisons between sample pairs.

The RNABC pipeline resulted in 51 out of the 57 paired samples matching on predicted CITBCMST subtype, while the remaining six samples were mismatches. For the six discordant samples, additional analyses were performed to examine the reason for the mismatch. A CITBCMST prediction for each of six samples was performed and the results visualized (Fig. 2). In the case of ‘HER2‐15', ‘HER2‐19', ‘LUMA‐26', ‘LUMA‐31', and ‘LUMB‐03', the errors are very small since the samples are predicted to be mixed using one of the data types and core using the other data type (confidence mismatches). In these five cases, the Spearman's Rho values for the two data types are above 0.95 and the points are very close in the PC space as shown in Fig. 2. In the final case, ‘LUMA‐04', the distance between the two points in PC space is more substantial (class mismatch). When investigating this further, there were no signs of poor data quality on either the microarray or RNA‐Seq level; however, the Spearman's Rho between the microarray and RNA‐Seq data is 0.9152, which is the lowest correlation of all the paired samples. Furthermore, for the 375 probes actually included in CITBCMST classification, the Spearman correlation is only 0.8337. The problem with a lower Spearman correlation for the 375 probes is not a general trend across the samples, so it would appear that this sample is an outlier case, in which concordance between the two data types is low for reasons unexplained by standard QC measures.

### 3.3 Validation of the RNABC pipeline on the RH dataset

**Figure 3:**
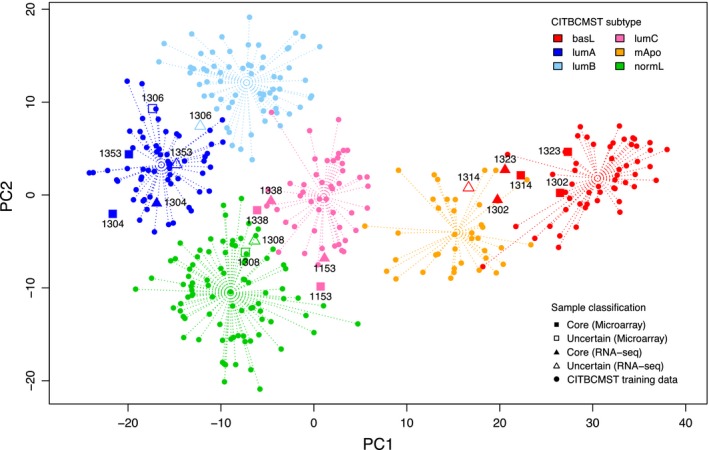
PCA plot of the CITBCMST training data (circles) and the nine samples of the RH dataset from both microarray (squares) and RNA‐Seq (triangles) data. Each sample point is labeled by a number to allow for direct comparisons between sample pairs.

To ensure more general applicability, a second dataset of nine samples was used to validate the performance of the pipeline. Beyond these being data generated at our clinic, validating on a smaller dataset is relevant since in clinical settings data are typically submitted in smaller batches of samples, which may affect the results of the batch correction step. For the RH data, the Spearman's Rho between the data types after RNABC transformation was 0.9734 (0.5722 before running the RNABC pipeline) and seven out the nine samples were predicted to be of the same subtype for both data types (Fig. 3). The two mismatches are both confidence mismatches: one sample was classified as lumA using microarray data and lumAB mixed based on RNA‐Seq data, and the other case is a switch from basL core to basL‐mApo mixed. Such minor discrepancies between data types in the subtyping of borderline cases can arise from minor variations, and we categorize the mixed cases as inconclusive for downstream clinical decision‐making regardless.

### 3.4 Comparison with other methods

**Figure 4:**
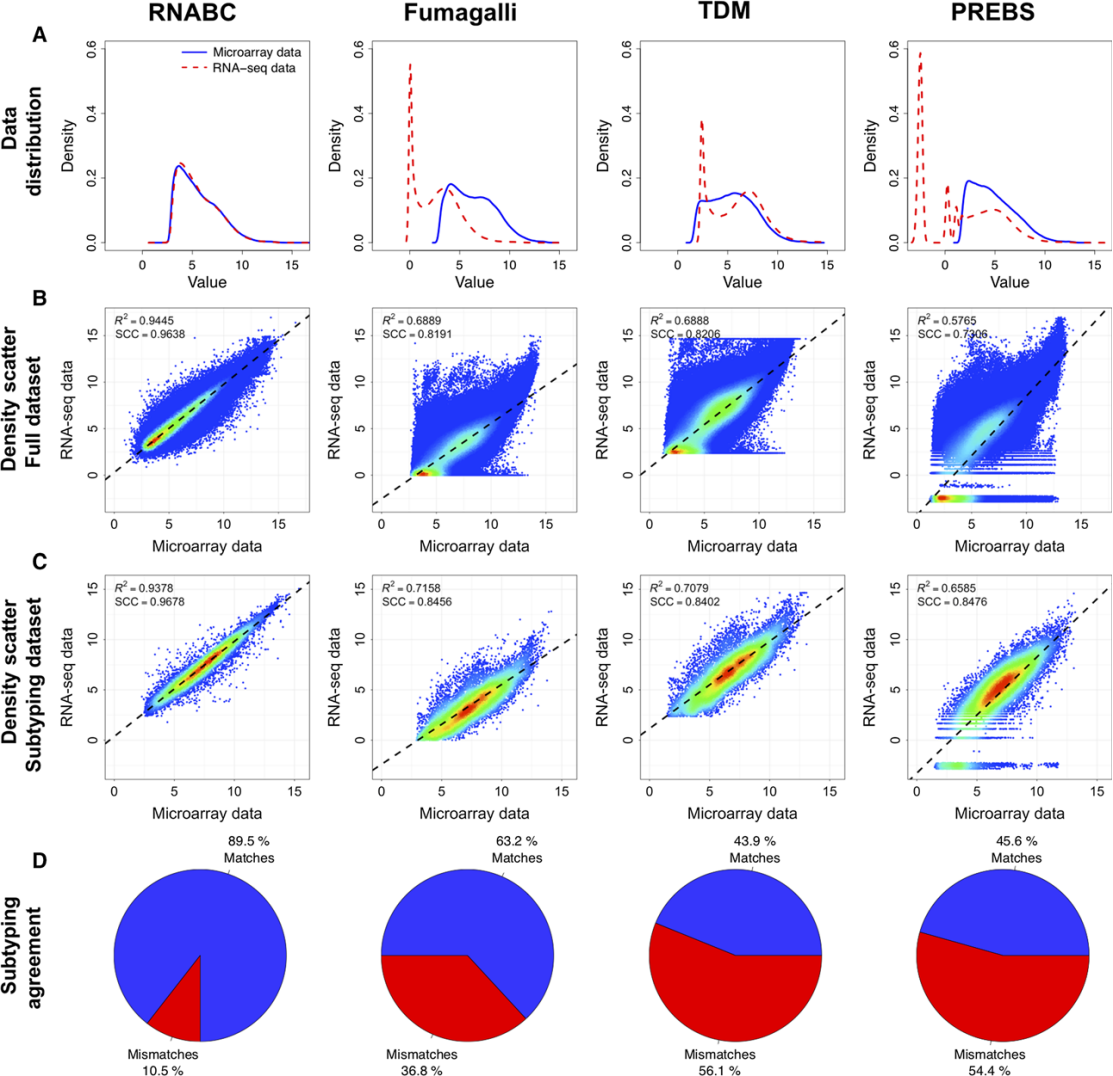
Comparison of RNABC and three other methods for comparing microarray data and RNA‐Seq data applied on the Bordet dataset. All plots represent the data after transformation. Each column represents a method, and the rows, from top to bottom, represent (A) a density plot of the distribution of the two data types across all 57 patients including all available probe sets/genes, (B) a density scatter plot for expression values for all available probe sets/genes, and (C) a density scatter plot for the probe sets/genes used in the actual subtyping with CITBCMST. The black dashed lines in B and C represent the least squares regression line, and the *R* ^2^ value for this line is printed in the top corner along with SCC for the datasets. (D) Percentages of matches and mismatches when comparing the CITBCMST prediction for microarray and RNA‐Seq data. For RNABC and PREBS, the total number of probe sets was 54 613 (all probe sets from the Affymetrix HG‐U133 Plus 2.0 Array except for the control probe sets) and the number used for subtyping was 375. For Fumagalli and TDM, the total number of genes was 18 734, and 238 and 234 were used in subtyping, respectively.

The performance of the three other methods for comparing microarray data and RNA‐Seq data was also assessed on the Bordet dataset. The results of three methods and the RNABC pipeline are summarized in Fig. 4. The Spearman's rank correlation is fairly good in all cases with values of 0.9638, 0.8191, 0.8206, and 0.7306 for the RNABC, Fumagalli, TDM, and PREBS methods, respectively, which means that the data are comparable in a general sense, but as evident from Fig. 4, using RNA‐Seq data without further processing as input to a prediction algorithm trained on microarray probe intensities yields suboptimal results. In terms of *R* ^2^, Spearman's Rho, and subtype matches, the RNABC pipeline proved superior to all other tested methods.

Also worth noting is that the runtime of the RNABC pipeline is a fraction of the other three tested methods, mostly owing to the speed of the kallisto algorithm. Not only does kallisto eliminate the need for traditional read mapping algorithms in the pipeline as reads are pseudoaligned directly against the probe sequences, but postprocessing (normalization and batch correction) is also near‐instantaneous, bringing the runtime down from hours to minutes per sample on a standard laptop computer.

## 4 Discussion

### 4.1 Discordance between subtyping using microarray and transformed RNA sequencing data

Some of the samples are almost equally close to two subtypes. Because of this uncertainty, these borderline cases receive a less meaningful class assignment than the core samples clearly belonging in a single subtype. Even minor differences in true and estimated probe abundance can contribute to class switching in the mixed cases, and clinically speaking, these classifications are not used. Samples that do not fit well into any of the subtypes also represent classifications without clinical utility. Both the outlier and the mixed cases point out a weakness of the entire subtyping paradigm in precision medicine. Every cancer patient is unique and even two patients that are predicted to be of the exact same subtype may be quite different on a molecular level. This lack of robustness should certainly be improved on, but this should be addressed by improving subtyping methods as well as patient stratification and treatment in general.

### 4.2 Importance of pipeline components

The RNABC pipeline consists of three main components: the mapping of RNA‐Seq reads to probe set target sequences, quantile normalization to the CITBCMST training dataset, and batch correction to the CITBCMST training dataset. The first step, the mapping of the RNA‐Seq reads directly to the probe set target sequences, allows for subtyping including all the 375 probe sets used as the original CITBCMST subtype signatures. This makes the subtyping more robust, than applying the CITBCMST classifier on the 256 HUGO symbol signature. With the goal of reproducing the microarray‐based subtyping from the RNA‐Seq data, both quantile normalization and batch correction are essential components of the pipeline. Probe set mapping alone results in no matches, as a result of poor correlation between microarray and RNA‐Seq data (*R* ^2^ = 0.0119, SCC = 0.6982), while quantile normalization alone following probe set mapping results in five matches (*R* ^2^ = 0.5540) in the CITBCMST subtyping for the Bordet dataset, and batch correction following probe set mapping yields only mismatches (*R* ^2^ = 0.0118). These results indicate that the application of mapping and both postmapping steps is necessary to ensure high accuracy of the classification. Interestingly, each of the two postmapping steps alone yields Spearman's rank correlation coefficients (SCC) of 0.6977 and 0.8796, respectively, which corresponds well to the observation in section 3 that a near‐identity relation is necessary to reproduce microarray‐based subtyping results. Finally, it should be emphasized that high‐quality data are paramount for obtaining accurate subtyping results, meaning that thorough QC is recommended before running this (or any other) pipeline.

### 4.3 Data transformation and batch correction

In this study, we transform RNA‐Seq data to match the distribution of the entire CITBCMST training dataset, which contains 355 samples of six different subtypes. This means that transforming the test data to the entire CITBCMST training data distribution can potentially introduce errors if the test data do not contain all six subtypes. Additionally, considering that samples in the clinical setting might be run individually, there is an even higher risk of transforming the data incorrectly. We recommend that samples always be run in batches for clinical applications. Alternatively, if it is not possible to obtain a balanced sample cohort, an option is to run the ComBat batch correction while accounting for covariates such as receptor status. The receptor status for all samples in the CITBCMST training dataset is available in the RNABC Bitbucket repository.

### 4.4 CITBCMST subtyping

The CITBCMST subtyping algorithm is only one of numerous breast cancer subtyping algorithms, and additional algorithms exist for other cancer types. This microarray‐based subtyping scheme was implemented in our laboratory and enabled consecutive molecular subtyping for informing clinical decision‐making, as well as a full transcriptome for downstream explorative analyses. There is, however, room for improvement of the algorithm, and we tweaked several methodological steps in this study. Most notably, the standard implementation does not perform batch correction, which we and others have shown drastically improves the ability to compare datasets. The recalculation of the PCA with the test set is also somewhat suboptimal (although strictly an aesthetic parameter) as each run will produce a different plot. Instead, we suggest projecting new samples in to a PC space precalculated on the training data, in order to ensure consistency in the output. In general, the robustness of the algorithm could also be improved upon. For this, a complete reworking of the subtyping scheme is probably necessary—preferably using RNA‐Seq data as a starting point. Until such time, our RNABC pipeline enables the utility of legacy microarray‐based subtyping methods.

### 4.5 Future perspectives

It should be noted that many different breast cancer subtyping schemes exist besides CITBCMST. The choice of subtyping scheme is not standardized, and the arguments for using one or the other may be based on experience with different approaches in a particular clinic. The lack of robustness of many existing subtyping methods calls for creating entirely new schemes for cancer subtyping based on high‐throughput sequencing, possibly including biomarkers from other omics data types, and ideally some form of patient immune profiling as well. The subtypes of the future algorithms can of course remain the same, and the training data could be switched using paired samples of training data, possibly with multiple replicates to ensure reliability. Having clinics phase out microarray analysis altogether requires strong scientific evidence that RNA‐Seq‐based subtyping can perform equally well or better than current methodologies. Despite the current challenges, more studies demonstrating the utility of RNA‐Seq are continuously conducted indicating a promising future for RNA‐Seq in clinical medicine. Hopefully, the increasing use of RNA‐Seq and this contribution to its utility will lead to new insights and inspire novel treatment options.

## 5 Conclusion

Molecular subtyping of cancer for clinical decision‐making is common practice for many cancer types. The majority of these tools are built from DNA microarray profiling of large cohorts of patients—in many cases through massive collaborative efforts—and the resulting classifications serve as international clinical standards. Gene expression profiling is to an increasing degree being performed using RNA‐Seq, but while the two data types are comparable in nonparametric analyses of transcript abundance, RNA‐Seq count data are not directly compatible with established probe intensity‐based subtyping methods.

We here present a method that enables fast and accurate subtyping of tumor samples for which gene expression is measured using RNA‐Seq. We tested our method on 66 breast cancer samples for which we have measured transcript abundance using both microarrays and RNA‐Seq, and were able to achieve near‐perfect correlation between probe intensities from microarrays and the probe‐level expression inferred from RNA‐Seq data (*R* ^2^ = 0.9445, Spearman's ρ = 0.9638) and near perfectly matched breast cancer subtype assignments from the microarray data using the corresponding RNA‐Seq data. We compared our method to three state‐of‐the‐art methods for comparison of the two data types and outperform them all significantly, as no existing tool is able to generate data comparable enough to enable accurate clinical subtyping. Furthermore, since we utilize pseudoalignment directly to the probe sequences, we circumvent traditional read alignment and thereby cut the processing time down from more than 3 h to < 3 min per sample on a standard laptop. The tool is freely available at https://bitbucket.org/cbligaard/rnabc/.

## Author contributions

LRO and MR conceived and designed the project. MR and FCN acquired the data. CBP and LRO analyzed and interpreted the data developed the computational framework. CBP and LRO wrote the manuscript.
